# Temporal and Geographic variation in the validity and internal consistency of the Nursing Home Resident Assessment Minimum Data Set 2.0

**DOI:** 10.1186/1472-6963-11-78

**Published:** 2011-04-15

**Authors:** Vincent Mor, Orna Intrator, Mark Aaron Unruh, Shubing Cai

**Affiliations:** 1 Department of Community Health and Center for Gerontology & Health Care Research, Brown University Medical School, Box G-S121, Providence, Rhode Island, USA; 2 Center for Gerontology & Health Care Research and, Department of Community Health, Brown University Medical School, Box G-S121, Providence, Rhode Island, USA; 3 Department of Community Health, Brown University Medical School, Box G-S121, Providence, Rhode Island, USA

## Abstract

**Background:**

The Minimum Data Set (MDS) for nursing home resident assessment has been required in all U.S. nursing homes since 1990 and has been universally computerized since 1998. Initially intended to structure clinical care planning, uses of the MDS expanded to include policy applications such as case-mix reimbursement, quality monitoring and research. The purpose of this paper is to summarize a series of analyses examining the internal consistency and predictive validity of the MDS data as used in the "real world" in all U.S. nursing homes between 1999 and 2007.

**Methods:**

We used person level linked MDS and Medicare denominator and all institutional claim files including inpatient (hospital and skilled nursing facilities) for all Medicare fee-for-service beneficiaries entering U.S. nursing homes during the period 1999 to 2007. We calculated the sensitivity and positive predictive value (PPV) of diagnoses taken from Medicare hospital claims and from the MDS among all new admissions from hospitals to nursing homes and the internal consistency (alpha reliability) of pairs of items within the MDS that logically should be related. We also tested the internal consistency of commonly used MDS based multi-item scales and examined the predictive validity of an MDS based severity measure viz. one year survival. Finally, we examined the correspondence of the MDS discharge record to hospitalizations and deaths seen in Medicare claims, and the completeness of MDS assessments upon skilled nursing facility (SNF) admission.

**Results:**

Each year there were some 800,000 new admissions directly from hospital to US nursing homes and some 900,000 uninterrupted SNF stays. Comparing Medicare enrollment records and claims with MDS records revealed reasonably good correspondence that improved over time (by 2006 only 3% of deaths had no MDS discharge record, only 5% of SNF stays had no MDS, but over 20% of MDS discharges indicating hospitalization had no associated Medicare claim). The PPV and sensitivity levels of Medicare hospital diagnoses and MDS based diagnoses were between .6 and .7 for major diagnoses like CHF, hypertension, diabetes. Internal consistency, as measured by PPV, of the MDS ADL items with other MDS items measuring impairments and symptoms exceeded .9. The Activities of Daily Living (ADL) long form summary scale achieved an alpha inter-consistency level exceeding .85 and multi-item scale alpha levels of .65 were achieved for well being and mood, and .55 for behavior, levels that were sustained even after stratification by ADL and cognition. The Changes in Health, End-stage disease and Symptoms and Signs (CHESS) index, a summary measure of frailty was highly predictive of one year survival.

**Conclusion:**

The MDS demonstrates a reasonable level of consistency both in terms of how well MDS diagnoses correspond to hospital discharge diagnoses and in terms of the internal consistency of functioning and behavioral items. The level of alpha reliability and validity demonstrated by the scales suggest that the data can be useful for research and policy analysis. However, while improving, the MDS discharge tracking record should still not be used to indicate Medicare hospitalizations or mortality. It will be important to monitor the performance of the MDS 3.0 with respect to consistency, reliability and validity now that it has replaced version 2.0, using these results as a baseline that should be exceeded.

## Background

Originally implemented in 1990 and mandated for nationwide use in response to the Nursing Home Reform Act of 1987, the Resident Assessment Instrument Minimum Data Set (MDS) 2.0 has been in use since 1997 and computerized into a national repository since October of 1998[1,2]. Initially designed as an instrument to summarize a detailed clinical assessment as a prelude to care planning, it was not long before its use was adapted for use in case-mix reimbursement to set daily payment rates for both Medicare and the Medicaid programs in nearly 40 state Medicaid programs[3,4]. This was followed by the creation and public reporting of quality measures based upon facility aggregates of selected MDS data items[5-7].

As a result of these expanded applications, the system of records governing the RAI became increasingly complicated. Under the original legislative mandate, the Centers for Medicare and Medicaid Services (CMS) required that a comprehensive resident assessment be completed within 14 days of admission to the facility. This assessment was to be completed at least quarterly thereafter, meaning that admissions for very short term stays would not have a documented assessment since it was not required[1,8]. A discharge record was introduced around the time that computerization was mandated in 1998. Specialized Medicare assessments were introduced with the Skilled Nursing Facility Prospective Payment System in order to document patients' level of need for care and rehabilitation within a few days of admission and at regular intervals until discharge, or upon resuming the standard schedule for long stay residents no longer covered by Medicare [9].

The increased complexity along with the requirement for submitting computerized MDS records meant that even small facilities had to purchase computers and software or make arrangements for data entry to transmit their data to the central CMS repository. Larger facilities and multi-facility chains purchased or developed specialized software with a wide variety of capabilities, some automatically generating resident care plans so facilities were in compliance with regulations, others that automatically updated unchanged fields for quarterly assessments and still others that checked for data internal consistency[10]. For facilities that engaged in data based quality improvement efforts, there was the possibility for checking the accuracy and consistency of their data, but there is little evidence that this was a common practice[11,12].

Field testing of the MDS and the quality measures revealed generally good levels of inter-rater reliability among those facilities that agreed to participate in these large and demanding research studies[13-15]. However, numerous smaller studies exploring the validity of the data elements that make up some of the quality measures that are publicly reported by CMS revealed problems with the validity of the indicators and variability in how the data elements are recorded[16,17]. It should be noted, however, that even studies that found a poor match between MDS items and research tools or medical records, found reasonable correlations between average MDS data and average research instrument scores at the facility level[18]. Other investigators, using the MDS data for epidemiological and health services research studies have found that, in aggregate, the data behave as expected with respect to the performance of summary scales and the predictors of hospitalization and mortality[19-23]. While the evidence suggests that MDS data can perform as expected in research studies, selected quality measures have been shown to have poor sensitivity in measuring quality[16,18]. Indeed, the quality of the MDS data has been shown to have considerable inter-facility variation, even amongst facilities that volunteered for a study of their quality of MDS assessment[15].

With the recent interest in pay-for-performance the reliability of the MDS discharge records in indicating hospitalizations and death is becoming important, yet the completeness and validity of these have not been examined empirically. Possibly this is due to the sophistication required to differentiate between types of MDS assessment records and to link admissions and continuing stay records to discharges, particularly since the definition of a discharge can be ambiguous.

In spite of ongoing questions about the reliability and validity of the MDS data and the related quality measures being publicly reported, CMS and the industry are committed to an MDS rooted in resident assessment which is also to be used for both payment and quality monitoring purposes. Indeed, a revised version of the MDS more focused on the "resident's voice" was tested and refined for several years and implemented in October, 2010[24,25]. This version requires staff to directly ask residents questions if they are able to respond while at the same time retaining many of the features of the MDS 2.0 that were used to calculate the case-mix measures necessary to determine facility reimbursement levels. While it is highly desirable to incorporate the residents' direct responses into the assessment process, the proportion of residents able to respond directly to questions is likely to vary substantially across facilities, introducing yet another level of complexity into the interpretation of the data[26].

Now that the MDS 2.0 is being replaced by the MDS 3.0, it is appropriate to consider selected aspects of the level of internal consistency achieved over the course of a decade of "real world" use along with the alpha reliability and validity of some of the key scales and measures that have been constructed and widely used for reporting and research. The purpose of this paper is to examine the reliability and predictive validity of the MDS 2.0 and how it has varied over time, and geographically. As has been well documented by researchers working with Medicare claims data, all administrative data systems have weaknesses and each change in the regulations underlying their use is likely to alter some aspect of these data in meaningful ways. After over a decade of use, the MDS 2.0 has reached relative maturity. In several years, it will be useful to compare the performance of the MDS 3.0 on some of the parameters we present here in order to assess its development and better understand its strengths and weaknesses.

## Methods

### Overview

We used person level linked MDS and Medicare denominator and all institutional claims including inpatient (hospital and skilled nursing facility) claims files for all Medicare fee-for-service beneficiaries residing in U.S. nursing homes during the period 2000 to 2007. We first documented the "completeness" of the MDS record relative to the "gold standard" Medicare claims and death records by estimating the proportion of skilled nursing facility (SNF) stays with a corresponding MDS and the proportion of hospitalizations and deaths based upon the MDS discharge record that agreed with Medicare information. We then compared diagnoses on the hospital claim with those on the MDS following admission from hospital. We also examined the rate of internal consistency of various MDS items that clinically should be highly consistent and finally examined the *alpha *reliability of commonly used MDS based scales and their association with subsequent mortality. We used the Residential History File (RHF) methodology to compare the discharge locations specified on the MDS discharge record with locations recorded at the same time on the RHF[27,28]. The analyses undertaken for this paper were done under the rubric of a Program Project grant from the National Institute on Aging "Shaping Long Term Care In America" [AG #27296] and the data used were assembled under a Data Use Agreement from the Centers for Medicare and Medicaid Services (CMS) (#18900) following review of the data base security protocols by the CMS Privacy Board and the Brown University Institutional Review Board.

### Sample

Our denominator included all persons with either an MDS assessment or a SNF claim. Most analyses were conducted only on nursing home residents who were age 65 or over. We used an algorithm to identify unique individuals in the stream of MDS records by matching the Medicare Health Insurance Claim number (HIC), Social Security Number, sex and date of birth. The more MDS records a person had with comparable matching variables, the more likely the records really pertained to a unique individual rather than reflecting a coding error. Thus, the longer the observation period per Medicare beneficiary, the more likely we were to have an identifier that matched to the Medicare enrollment record. We calculated match rates as the proportion of MDS identified individuals matched to the CMS denominator file's HIC, gender and date of birth.

After linking MDS and Medicare denominator files, we derived a Residential History File (RHF) for all the residents in the cohort. Described in detail elsewhere, the RHF is a sequential, longitudinal record that tracks residents' changing location over time; for example, transitions between hospital and nursing home. The RHF is created from all Medicare part A claims (inpatient hospital, Skilled Nursing Facility, out-patient, home health and hospice) and MDS assessments linked chronologically per individual according to their timing. In cases of overlap of services (for example, inpatient and SNF) a data hierarchy based on data reliability is used to infer location. The RHF forms a personal history[28]. We excluded Medicare beneficiaries who were members of a Medicare Managed Care plan since those individuals' utilization events are not captured in the standard Medicare fee for service claims systems.

From the population of Medicare beneficiaries with at least one MDS record over the period 2000 to 2006, we developed two different analysis samples, each stratified by year. First, we separately linked hospitalization claims of individuals who had not had a prior MDS record, with their first MDS record immediately following their discharge from the hospital. This allowed us to associate diagnoses listed in the Medicare hospital claim to the MDS admission assessment record. In addition to comparing diagnoses across the Medicare hospital claim and the MDS, we used the same admission MDS record to examine the internal consistency of selected parts of MDS items and the inter-item consistency of MDS items that have been reported as representing multi-item scales. Secondly, we examined all MDS discharge tracking forms and inpatient admissions from nursing homes identified in the RHF in order to determine the likelihood that an MDS discharge records matched to a hospitalization record, and whether a hospitalization record was preceded by a MDS discharge. Since all nursing home admissions under the Medicare Skilled Nursing Facility (SNF) benefit should have at least one MDS to determine their RUGS payment level, we tested the extent to which this was true in the data. Lastly, we examined whether deaths in the nursing home, as identified in the RHF, had a corresponding MDS discharge tracking form indicating death, and whether all MDS discharge tracking forms coded as 'death could be validated in the RHF.

### Measures

We present the results of three different sets of analyses using data from both the MDS records and the Medicare claims. For the purpose of these analyses we view the Medicare claims as the "gold standard" both for the diagnoses and the dates of service from inpatient and SNF claims. From Medicare claims we use the ICD-9 diagnoses coded on the hospital claim, which allows for up to 10 different discharge diagnoses and an admission diagnosis. We rely heavily on the dates of admission and discharge from the hospital, since, in determining the "validity" of the MDS discharge destination of hospital, we examine both the exact date match of the MDS discharge date and the Medicare hospital claim admission date.

We compare the presence of selected diagnoses on the hospital discharge claim to those indicated on the MDS admission record, which uses a "check box" approach rather than ICD-9 coding. While the MDS form does allow nursing home staff to write in actual ICD codes, those fields are rarely complete. The groups of ICD-9 codes on the Medicare claim were contrasted with the presence of a positive "check box" on the MDS for an appropriate diagnosis (e.g. heart failure on the MDS was equated to heart failure and cardiomegaly - ICD-9 codes 398.91, 402, 404, 428). In selecting which MDS "check boxes" to compare to hospital discharge diagnoses, we focused on those least ambiguous with respect to the cross-walk with ICD codes and built upon our earlier work in this area[29].

Next, relying only upon data in the MDS admission assessment, we examined the internal consistency of selected pairs of items within the MDS that should logically correspond to one another. For example, residents who have no dependency in any Activities of Daily Living (ADL) should not be bed-bound, hemiplegic or unable to move in their own bed independently. The rationale for this exercise is to estimate the extent to which there is obvious "noise" in the MDS as used in the real world. However, we moved beyond these most obvious comparisons to determine whether those with the most severe Cognitive Performance Score (CPS) had a diagnosis of dementia and/or Alzheimer's disease[19]. While not all severely cognitively impaired residents should be demented, particularly immediately after a hospitalization, a dementia disorder should be the dominant reason for cognitive deficits measured in the CPS. We went somewhat further afield and compared a checked arthritis diagnosis with a checked joint pain symptom on the MDS assessment, and a checked treatment with a diuretic and a checked edema symptom on the MDS. For each of these paired comparisons we calculated specificity, sensitivity and the positive predictive value (PPV) of each predicated relationship.

We tested the internal consistency, or the *alpha *reliability, of several multi-item scales that have been characterized in the literature based upon the MDS, using the same admission MDS. We did this for the total population of residents and also stratified the performance of the well-being, mood and behavior scales according to two functional measures: physical function above or below the median value of the long form of the Activities of Daily Living (ADL), and the mid-point (3 of a score of 0 to 6) of the Cognitive Performance Scale (CPS)[30]. The rationale for calculating the internal consistency of these items separately within these physical and cognitive functional strata is because the variability of measures such as depression, pain, social engagement, etc. can be expected to differ across these strata.

Next, we tested the extent to which the presence of an MDS admission and discharge record correspond to the sequence of Medicare claims data, including dates of discharge to hospital on the MDS discharge record and dates of admission in the Medicare inpatient hospital claims. Since dates of service are frequently one day off in light of how dates of discharge and admission are treated (can't be billed for two inpatient services on the same day), we allowed a three day non-exact match tolerance in determining the rate of "exact correspondence" between MDS events and Medicare claims based events. Likewise, we examined the indication of death on MDS discharge tracking forms by comparing with deaths in the RHF that were within 2 days of a nursing home stay. Finally, we examined the frequency with which an MDS is filed during a SNF Medicare-paid nursing home stay, based upon the existence of a Medicare SNF claim as another means of assessing how much data are missing or incorrect (relative to Medicare claims) if one relies only upon the MDS records.

We examined the predictive validity of several composite measures of functioning and frailty that have been developed including ADL, CPS and the Changes in Health, End-stage disease and Symptoms and Signs (CHESS)[31,32]. All three were based upon admission MDS data and were used to predict one year survival using the vital status information included in the Medicare denominator file, regardless of whether the individuals remained in the nursing home. These analyses were stratified by age to test the independent effect of the frailty measures on the likelihood of surviving one year, controlling for age.

### Analytic Approach

We calculated the sensitivity, specificity and positive predictive value (PPV) of the MDS diagnoses relative to the "gold standard" of the Medicare hospital claims diagnosis for each year and separately for each state, allowing us to test both the effect of time and geography. We calculated the *alpha *internal consistency, reliability measure using Chronbach's alpha[33]. All analyses were performed using SAS version 9.2 and STATA 10.0.

## Results

As can be seen in Table 1 match rates in all years (1999-2007) among residents who were 65 years old or older exceed 95% with slightly higher numbers pertaining to earlier years, precisely because we had more data to correct "errors".

**Table 1:** Match Rate between MDS records and Medicare's Enrollment File from the CMS MDS Registry by calendar year

Year	# with an MDS 65 and over	# Match MDS-Medicare 65 and over	Percent match 65 and over
1999	2,895,632	2,795,080	96.53

2000	2,873,778	2,772,442	96.47

2001	2,907,589	2,787,048	95.85

2002	2,892,291	2,782,532	96.21

2003	2,876,013	2,778,992	96.63

2004	2,880,140	2,787,829	96.79

2005	2,926,867	2,844,360	97.18

2006	2,932,121	2,841,366	96.90

2007	2,945,183	2,858,947	97.07

Table 2 presents a sample description of the new admissions entering US nursing homes in 2000, 2002, 2004 and 2006 based upon the Medicare denominator file information including date of birth, gender and race along with several summary measures from the admission MDS records. As can be seen, the average age at first nursing home entry is 81 years and this has remained fairly stable over the course of the decade. On the other hand, we are seeing a substantial increase in the number of diagnoses reported on the Medicare hospital claim preceding patients' first admission to a nursing home. The steady increase in the ADL dependency of the admission population is consistent with the number of diagnoses, but we don't see that increase in impairment reflected in either the level of cognitive impairment or acuity as reflected by the CHESS score. Interestingly, while the age distribution didn't change much over the period (~ 20% for those 65-74 and over 30% for those over 85), the proportion of male admission did increase by over 2 percentage points.

**Table 2:** General Characteristics of Population by Year

	2000	2002	2004	2006
	*n *= 790,227	*n *= 790,617	*n *= 773,746	*n *= 718,555
**Variable**				

**Mean Age (± SD)**	81.1 ± 7.3	81.1 ± 7.3	81.0 ± 7.3	81.0 ± 7.4
**65-74**	20.3%	20.5%	20.7%	21.1%
**75-84**	45.7%	46.1%	46.1%	45.6%
**85+**	33.9%	33.4%	33.1%	33.3%
				
**Gender**				
**Male**	33.4%	34.5%	35.1%	35.7%
**Female**	66.6%	65.5%	64.9%	64.3%
				
**Mean number of Diagnoses (± SD)**	7.0 ± 2.2	7.4 ± 2.1	7.7 ± 1.9	8.0 ± 1.8
**1-2**	3.8%	2.7%	1.9%	1.3%
**3-4**	12.5%	9.7%	7.4%	5.8%
**5-6**	20.5%	17.3%	14.4%	11.8%
**7-8**	21.6%	20.8%	18.8%	16.4%
**9**	41.6%	49.6%	57.5%	64.7%
				
**Cognitive and functional measures**				
**Long Form Activity of Daily Living Scale (± SD)**	14.1 ± 7.0	14.5 ± 6.9	14.8 ± 6.7	15.4 ± 6.2
**Cognitive Performance Scale (± SD)**	1.5 ± 1.7	1.5 ± 1.7	1.4 ± 1.6	1.4 ± 1.6
**CHESS (± SD)**	1.8 ± 1.1	1.8 ± 1.1	1.8 ± 1.1	1.8 ± 1.0

SD: standard deviation

We examined the completeness of the MDS relative to those admitted under the SNF benefit and found that no MDS of any type was found for 9.4% of SNF episodes in 2000 but this decreased to only 5% by 2006. Nonetheless, only 82% of these assessments were of an admission type (admission, 5-days, or re-admission) which one would expect since this is required.

Next, we found that among deaths in the Medicare files, 84.4% had an MDS discharge record indicating death at discharge and an additional 12.4% had a discharge to some other location (generally hospital), meaning that fewer than 4% of deaths were missing a discharge record and by 2006 this was only 2.6%. Amongst the population of cases with an MDS discharge record, we found that by 2006 94.8% of individuals with a discharge had died according to the Medicare files, or just over 5% of MDS discharges had a death filed incorrectly.

**Figure 1:**
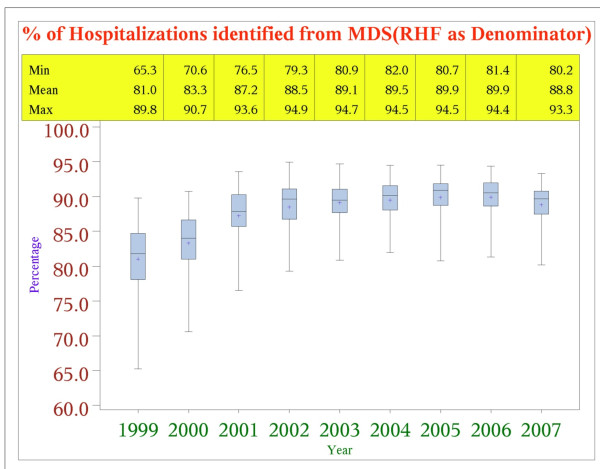
**Percent of Medicare Claims Hospitalizations Identified from MDS Discharge Records: 1999-2007**. (N = 4,395,102).

Figure 1 presents the proportion of Medicare paid hospitalizations from nursing home of fee-for-service beneficiaries which were recorded by an MDS discharge tracking form, indicating discharge to a hospital, within 3 days of the inpatient admission day (either before or after). As can be seen, the rate of MDS discharge records reporting a hospital discharge rose from about 81% in 1999 to almost 90% by 2006, with the largest improvement occurring around 2001.

We examined all the MDS discharge tracking forms indicating discharge to hospital. The number of discharge tracking forms to hospital rose from about 1.1 million in 1999 to almost 1.5 million in 2006. Throughout the period, we identified Medicare inpatient claims for only 78% of the discharge records. The timing of the Medicare claim was on the same day as the MDS discharge record for about 75-79% and within 1-7 days for about 15-18%. The remaining 6-10% had a discharge MDS that was filed during their hospital stay or even after the hospital stay ended.

Among the 22% of MDS discharge tracking forms without an associated Medicare hospitalization claim, between 81% and 87% were in a nursing home according to the Residential History File, and an increasing number were in the emergency department (5% in 1999 increasing to 8% in 2006) or under observation days in the acute hospital (7% in 1999 increasing to 9% in 2006). The proportion of MDS discharge assessments indicating hospitalization that could be matched to inpatient claims varied across states, for example, between 66.3% in Arizona to 85.6% in Kansas in 2005.

Table 3 presents the results of comparing the MDS diagnoses on the admission assessment with those on the Medicare hospital claim discharge diagnoses for selected diagnoses. Presented are the PPV, the PPV inter-quartile range across the states and the sensitivity and specificity of the Claims based vs. the MDS based diagnoses. We conducted the analyses for all years between 2000 and 2006 but since the pattern of results was quite similar across all years, we only present the most recent year. Additional File 1 presents a summary of this information for all years of data. With a few exceptions, most of the diagnoses have PPV in excess of .6. Heart failure, diabetes and COPD/asthma/emphysema all had high PPV levels while Depression, stroke and any dementia had lower PPV levels. The PPV of Parkinson's Disease changed substantially over the study period, from .76 in 2000 to .60 in 2006, with a relatively high inter-quartile range but diabetes also declined over the period [see Additional File 1]. The sensitivity levels of the MDS to identifying "true" positives in the Medicare claims are similar to the PPV with certain exceptions but, specificity levels of the MDS diagnoses was high, meaning there is substantial agreement with respect to a diagnosis not being present. By and large, the inter-state variation as measured by the inter-quartile range for most of the comparisons is relatively small, particularly for those diagnoses with high levels of PPV.

**Table 3:** Positive Predictive Value of MDS based diagnosis relative to ICD-9 Diagnoses on the Medicare Hospital Claim for selected Diagnoses:

Medicare Hospital Claim Diagnoses	MDS Diagnoses	PPV	Sensitivity	Specificity	PPV inter-state Inter-quartile range
Any hypertension	Hypertension	0.61	0.65	0.61	0.038

Heart failure/Cardiomegaly	Heart failure	0.78	0.67	0.94	0.038

Cerebro-vascular Accident	Stroke/TIA	0.32	0.70	0.90	0.064

Parkinson's disease	Parkinson's disease	0.60	0.69	0.99	0.090

Alzheimer's disease	Alzheimer's disease	0.66	0.53	0.98	0.076

Brain degeneration	Any-type dementia	0.32	0.72	0.86	0.058

Asthma/COPD/emphysema	Asthma/COPD/emphysema	0.80	0.75	0.95	0.036

Any pneumonia	Pneumonia	0.63	0.60	0.96	0.064

Depressive disorders	Depression	0.25	0.78	0.83	0.050

Diabetes mellitus	Diabetes mellitus	0.69	0.93	0.89	0.039

Any cancer	Cancer	0.55	0.51	0.95	0.076

Any anemia	Anemia	0.51	0.39	0.88	0.055

Any UT infection	Urinary tract infection	0.62	0.61	0.92	0.054

2006; N~718,555

**Figure 2:**
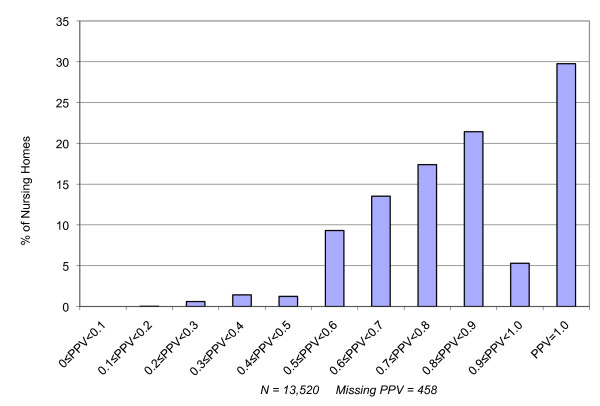
**Facility Variation in the Positive Predictive Value of Medicare Hospital Claims for Heart Failure and MDS admission assessments indicating a diagnosis of Congestive Heart Failure**. N = 13,520 Nursing Facilities.

To address the issue of inter-facility variation in the correspondence between Medicare hospital discharge diagnoses and MDS based admission diagnoses, we calculated the PPV at the level of the individual facility for facilities with a minimum of 100 admissions during the course of 2006. Figure 2 presents the distribution of facilities with various levels of PPV for the diagnosis of heart failure. As can be seen, most facilities with large numbers of admissions from hospital in the year have reasonably high PPV levels when comparing Medicare hospital claims diagnoses with diagnoses on the admission MDS. Indeed, nearly 70% of facilities have a PPV in excess of 70% for heart failure, reflecting the high average of .78 for heart failure in 2006. However, some facilities have quite low levels of PPV in spite of the fact that they have many admissions per year directly from hospital.

Table 4 presents the results of the internal consistency analyses comparing how well MDS items that should be logically related actually do correspond in terms of the positive predictive value, along with the inter-state variation in the positive predictive value of the association between the two variables. (See Additional File 2 for tables summarizing these statistics for all years of data.) All the ADL related items demonstrate very high levels of internal consistency that has been very consistent over time. Interestingly, the correspondence between having a Cognitive Performance Scale score of zero (no obvious signs of cognitive impairment or memory loss) and the presence of a check-box diagnosis of Alzheimer's disease or any dementia is quite high (>.95) and the inter-quartile range based upon state level average PPV levels is very small, suggesting comparably high rates of association across the country. Joint pain, vision, asthma/COPD/emphysema were only moderately associated with their respective criterion variables but cancer and chemotherapy and pressure ulcer care and pressure ulcers were reasonably highly associated.

**Table 4:** Positive Predictive Value and Internal Consistency of selected MDS items on the Admission MDS for 2006 and inter-quartile range across states

MDS "Gold"	MDS Diagnoses	PPV	Sensitivity	Specificity	PPV Inter-quartile Range
ADL ≠ 0	Hemiplegia	0.99	0.05	0.99	0.008

ADL ≠ 0	Bed-ridden	0.99	0.04	0.97	0.013

ADL ≠ 0	Bed mobility = 0	0.97	0.00	1.00	0.000

ADL ≠ 0	Terminal prognosis	0.99	0.02	0.99	0.014

ADL ≠ 0	Pressure sore stage 3-4	0.99	0.03	0.99	0.015

CPS ≠ 0	Alzheimer's disease	0.96	0.10	1.00	0.022

CPS ≠ 0	Vascular-type dementia	0.93	0.25	0.98	0.029

Visual impairment	Cataract	0.47	0.05	0.99	0.115

Edema	No dehydration	0.38	0.99	0.01	0.139

Joint pain	Arthritis	0.42	0.44	0.85	0.071

Cancer	Chemotherapy	0.57	0.05	1.00	0.137

Any ulcer	Ulcer care	0.98	0.73	1.00	0.008

Edema	Diuretic received	0.55	0.04	0.98	0.132

N~718,555

Researchers have constructed various multi-item scales from the item set in the MDS. Using data from the new admission cohort in 2006, we calculated the standardized Chronbach's alpha reliability (inter-item consistency) coefficient for the ADL scale, social engagement scale, the mood (depression) scale, the behavior problem index and the pain scale[20,34,35]. We first tested Chronbach's alpha for the entire population of new admissions in 2006. Next, separately for strata defined based upon the median ADL score and the mid-point of the CPS, we calculated Chronbach's alpha for the four sub-groups since we anticipated that such different patient groups might exhibit different patterns of correlation among the items in the scales. The standardized reliability coefficient for the long form ADL scale was .90 (data available from authors upon request), the Social Engagement Scale was .63, the mood scale was .65, the behavior scale was .53 (although without the manic depression diagnosis item it is .66) and pain was .5.

Table 5 presents the results of the stratified analyses, revealing standardized alpha coefficients for the sub-populations defined on the basis of ADL and CPS groups. As can be seen, they are relatively close to those of the overall population and close to the levels reported by the scale developers. The difference between the response patterns among the cohorts defined by the intersection of high and low ADL and CPS is not large, but reveals interesting patterns. Consistent with the expectation that staff have more difficulty assessing cognitively impaired residents, the standardized alpha coefficient for all scales is consistently lower among those with low CPS. The pattern with respect to high vs. low ADL performance is more subtle; while the low ADL cohort reveals lower reliability for social engagement and depression, the difference is quite small for behavior and seems to move in the opposite direction for pain intensity.

**Table 5:** Standardized Alpha Multi-Item Scale Reliability & Internal Consistency Stratified by Median Activities of Daily Living

Social engagement scale		
	ADL-high	ADL-low

CPS-high	0.62	0.60
		
CPS-low	0.60	0.59

**Mood scale**:		

	ADL-high	ADL-low

CPS-high	0.66	0.65
		
CPS-low	0.59	0.57

**Behavior scale**:		

	ADL-high	ADL-low

CPS-high	0.53	0.53
		
CPS-low	0.38	0.39

**Pain:**		
	ADL-high	ADL-low

CPS-high	0.49	0.52
		
CPS-low	0.47	0.49

**Figure 3:**
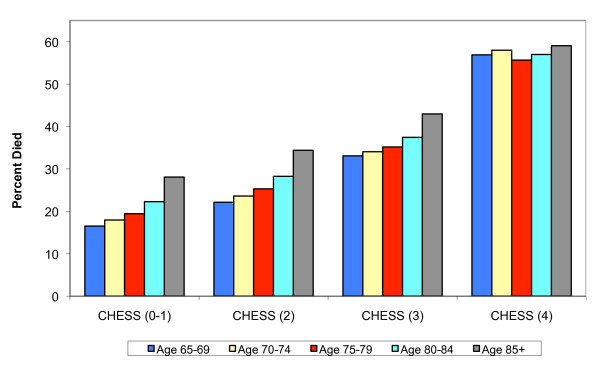
**One Year Survival by CHESS Score Level and Age**. N = 718,555.

Various "validity" studies of the MDS and its applications have been undertaken, often comparing "research" measurements done by clinicians or research assistants with information in the most recent MDS in the residents' files. Another approach to testing the construct validity of some aspect of the MDS is to examine the relationship between selected items and concepts which the literature and clinical practice tells us should be related to readily measured "objective" outcomes like death or hospitalization. We tested the "predictive validity" of the CHESS scale, which was designed as a composite measure of medical stability, frailty and clinical acuity, to predict mortality amongst frail elders in institutional settings[31]. We identified new admissions to US nursing homes in 2006 and observed them for at least one year, regardless of whether they remained in the nursing home, to determine whether they'd died according to the Medicare enrollment file. Figure 3 summarizes the relationship between quartiles of the CHESS scale and one year mortality, stratified by age upon admission. As can be seen, there is a doubling in the one year mortality rate among 85 year olds between the lowest and highest quartile of the CHESS scale, from around .30 to .60. Perhaps because this is a new admission cohort, many of whom do not remain nursing home residents but are re-hospitalized and die or return under hospice care, we see a strong monotonic effect of chronological age categories on one year mortality within quartiles of the CHESS score until the highest risk CHESS category is reached, at which point age doesn't appear to matter. We conducted similaer analyses of the predictive value of the long form ADL scale as well as the CPS and both were found to be strongly related to one year mortality, although not as strong as the CHESS since it was designed precisely to be predictive of survival.

## Discussion and Conclusion

We undertook a comprehensive data based approach to testing the consistency and utility of the MDS for administrative reporting and research uses. To do so, we used national Minimum Data Set Registry data merged with Medicare enrollment and inpatient claims files data covering 1999 through 2007 to assess the "validity" of the MDS record sequencing, diagnostic information as well as the internal consistency and validity of the MDS items and the clinical research scales that have been developed. Results warrant a fairly positive appraisal of the MDS. First, the match rates between MDS data and Medicare records exceeded 95% for the population 65 and over and there is very little inter-state variation. Second, in examining the completeness of the MDS data relative to Medicare records, we found reasonably high correspondence between discharge records and Medicare hospitalizations and deaths and found that most SNF stays have a corresponding MDS admission. However, there were many more hospitalizations according to the MDS discharge tracking record than were substantiated by Medicare records. Third, many of the "check box" diagnoses on the MDS correspond reasonably well (PPV> .7) to the gold standard of the Medicare hospital IDC-9 diagnoses, although without the obvious precision of an ICD code. Fourth, the data items within the MDS record expected to be internally consistent appear to be so (e.g. measures of physical functioning) with PPV > .95, and those where there is less expectation of agreement have lower, but still reasonably high levels of agreement. Fifth, the internal consistency of proposed multi-item scales included in the MDS were found to be excellent to moderate and relatively constant across very different groups of patients with respect to cognitive and physical functioning. Sixth, we validated a composite acuity and frailty score, the CHESS scale, and found it to be highly related to one year mortality based upon Medicare records, stratifying for age. The paragraphs below discuss the implications of these results for the broad scale use of the MDS for payment, quality monitoring and research and for those charged with monitoring the implementation of the new MDS 3.0 introduced in October of 2010.

Our finding that major diagnoses noted in the MDS are reasonably consistent with the diagnoses enumerated in the Medicare hospital claim replicates our finding from the early days of the MDS when only a few states were computerizing assessments[29]. The MDS manual stipulates that MDS diagnoses are those that affect treatment or function which is consistent with the instructions hospital coders adhere to under Medicare billing[36]. We did observe a large increase in the number of diagnoses listed on the hospital claim but it didn't really influence the observed PPV's, which we found to be relatively stable over time. The increase in hospital diagnoses occurred presumably because of changes in reimbursement policies and the expanded use of quality measures which may have pushed hospitals' coders to note increasingly specific diagnosis and procedure codes. Thus, our findings suggest that use of cardio-vascular disease, diabetes, Alzheimer's disease and several others will yield research results consistent with use of Medicare claims data. While there is some inter-facility variation in the PPV between the two sources of diagnostic information, by and large it appears reasonable to use MDS diagnostic data to predict outcomes like hospitalization, discharge home or even death.

Almost since the initial design of the MDS, disputes about the reliability, validity and accuracy of the data have raged[2,37-40]. While a number of large reliability trials consistently found moderate to excellent levels of inter-rater reliability between research and staff nurses, other studies have found poor correspondence between facility medical records, patient observations and the data in the MDS[14,15,41,42]. Some have noted substantial inter-state and inter-provider [43]variation in data quality and completeness and that facilities which participated in reliability studies differed substantially from those that didn't[15,44]. We didn't observe much inter-state variation in agreement rates suggesting that data on items' internal consistency and agreement with hospital diagnoses is reasonably strong and consistent across states. While we did observe some inter-facility variation in selected diagnoses, most facilities had high correspondence between hospital and MDS diagnoses. As importantly, we did find that summary scales derived from the MDS on ADL, mood, behavior, social engagement achieve excellent to moderate levels of alpha reliability, suggesting that these scales have measurement properties that permit their use in some types of research applications. Moreover, these scales seem to be based upon consistent patterns of inter-relationships among the MDS scale items in very different sub-populations since the alpha coefficients were consistently observed across very different clinical populations.

In light of our findings, how should the MDS data be used? The MDS was designed to document and guide a uniform resident assessment process for the purpose of developing a care plan[1]. As such, the clinical information should be used to guide individual clinical decisions. Clearly the MDS doesn't do that since the data are not updated in real time, rather only once a quarter to represent a snapshot in time of the resident's condition. The MDS wasn't supposed to replace the more dynamic medical record and nursing notes. When initially designed over 20 years ago, the acuity and risk of change in clinical status was much lower than among today's more clinically complex nursing home population. The question remains then - how good is this "snapshot" and what are the implications of using it for reimbursement, monitoring providers' quality performance or research?

Medicare and nearly 40 states' Medicaid programs currently use MDS data to apply some form of case mix reimbursement that increases payment rates as a function of the acuity and functional limitations of the residents[45-47]. Medicare uses MDS data to determine the exact payment to a given facility on behalf of a specific patient while most state programs apply case-mix adjustment at the aggregated level of the facility. Zinn and colleagues concluded that adopting this reimbursement model is associated with greater resident acuity, suggesting improved access for sicker residents or more aggressive "up-coding"[47]. The one research audit done to address this issue was done by the General Accountability Office. They found as much under as over coding of patients' conditions relative to nurses notes or research staff assessments, a finding that is consistent with an analysis of the directionality of inter-rater reliability errors in the MDS of nursing staff relative to research nurses[44]. Thus, use of the MDS for reimbursement is not substantially different from using Medicare hospital claims for the application of prospective payment rates, with respect to overall accuracy.

Since the early part of the last decade, the Centers for Medicare and Medicaid Services has been using MDS data to create and publicly report quality measures at the level of the facility, contingent upon there being a sufficient number of residents in the home. Such aggregated measures can tolerate a certain level of error particularly since the quality measures being used are not highly correlated[48]. There may be somewhat more systematic bias by state since even small differences in the directionality of the error within a facility or across facilities in a state can compromise the validity of quality measures substantially[44]. However, there are various problems with the current quality measures such as the stability of the measures, the lack of correlation amongst them and the limited level of risk adjustment that have a far greater effect on the meaningfulness and performance of the quality measures than the level of error in the data[49,50].

Using the MDS for research, policy evaluation and planning has the advantage of not requiring the same level of precision as is needed to justify a clinical decision about an individual resident nor even as definitive as should be necessary to publish the relative ranking of one home over another on a given quality measure. Furthermore, there are statistical means of "adjusting" out the idiosyncratic measurement error that can occur in some facilities and not others, still making it possible to examine the effect of states' policies on resident adjusted outcomes such as pain or ADL[51,52]. Evidence of the strong monotonic relationship between the CHESS scale and one year mortality among new NH admissions is clearly at least as strong as the Charlson Index or the Deyo-Elixhauser scale as applied to hospital discharge diagnoses. The existence of standardized physical, cognitive, emotional, social and behavioral functioning scales which are largely invariant across different types of patients provides further evidence of the utility of these data for research and policy applications.

### Implications for the MDS 3.0

The transition from MDS version 2.0 to 3.0 is a major change since many of the individual MDS items have been altered in important ways[25]. Most importantly under the new version the residents' perspective is supposed to be "heard" if at all possible. Although the requirement to interview the resident introduces a new and important feature into the MDS 3.0 data, it poses difficult measurement issues. Self-report and staff rating responses have been reported to vary due to the different perspective residents and staff bring to assessing most aspects of symptom and psycho-social experience and are only moderately correlated, at best[53]. Thus, constructs like pain, which have measurement limitations in the current version of the MDS, will present additional complexity in MDS 3.0 as a consequence of the separate voices, the patients' and the staff assessors', regarding the construct being measured. This additional heterogeneity introduces an expansive new research agenda for those interested in long term care and in basic measurement issues that are the building blocks of an increasing number of quality measurement initiatives. Thus, it will be critical to document and systematically characterize residents who can and cannot respond to questions and to monitor how that varies across facilities and over time since this aspect of the MDS 3.0 represents an important new kind of measurement challenge that must be considered in comparing the related quality measures

With the introduction of MDS 3.0 complete resident assessments have been required at discharge (not only filing a tracking form). This creates an opportunity to significantly improve the quality of the MDS discharge records and their timeliness. Our finding that facilities submit many more discharge records indicating hospitalizations than there are Medicare-paid hospitalizations, suggests that the MDS discharge records as currently completed and filed, should not be used as the basis for monitoring this outcome; if a Medicare hospital claim is present, an MDS discharge is likely to be present, but the opposite is not the case. Requiring that a discharge assessment of the patient be completed rather than merely documentation of the discharge might reduce the number of unnecessary discharge record submissions. All dynamic record systems require ongoing and careful monitoring to ensure standardization. Any greater specificity regarding when a discharge is recorded and submitted will be a great improvement over what exists today. Future research examining the completeness of these records relative to Medicare claims and enrollment files will hopefully reveal greater correspondence and completeness with relatively little inter-state or facility variation.

In summary, after years of use, our analyses covering virtually all MDS data completed in the US between 1999 and 2007 find that the MDS data cross-walk reasonably well with Medicare hospital claims diagnosis data, both with respect to the match rate and the validity of the MDS diagnoses. The MDS data are generally internally consistent and several of the multi-item scales based upon MDS items have reasonably good levels of internal consistency and reliability. On balance, the MDS data can be very useful for research and program planning and evaluation and the introduction of the MDS 3.0 offers considerable opportunities to improve the quality and completeness of some of the data. At the same time the implementation of MDS 3.0 creates additional analytic challenges precisely because it endeavors to introduce the patient's voice into the assessment system. Over the next several years as analysts work to establish data quality benchmarks as well as the quality measure distributions using the MDS 3.0, the data presented here on almost a decade of MDS 2.0 data provides a standard of consistency, reliability and completeness against which the MDS 3.0 should be compared.

## Competing interests

Vincent Mor, Ph.D. declares the following potential competing interests:

Dr. Mor serves as a member of several Centers for Medicare and Medicaid Services (CMS) Technical Advisory Panels relating to the measurement of nursing home quality based upon the data included in the MDS, both the previous 2.0 version as well as the newly introduced 3.0 version. Dr. Mor has received grant and contract funding from U.S. federal granting agencies, U.S. Foundations and private companies for research using the MDS matched to Medicare claims for the past decade. Dr. Mor is the Chair of the Independent Quality Committee for hcr-ManorCare, a U.S. nursing home chain for which he receives an annual retainer. Dr. Mor is a member of the board and a shareholder of PointRight, Inc., an information services company providing data quality and other services to the U.S. nursing home industry. Prior to submission for publication, the results of this study have not been reviewed by any funding agency or company with which Dr. Mor is associated.

Drs. Intrator and Shubing as well as Mr. Unruh declare that they have no competing interests.

## Authors' contributions

VM specified all the data analyses presented in this manuscript, assembled the initial draft contributions of the co-authors and finalized the drafts submitted as well as the revisions and responses to the reviewers.

OI supervised the data base construction, the analyses of the completeness of the MDS records relative to the Medicare enrollment and claims files and wrote the relevant sections of the Methods and results section, editing and contributing to all other parts of the manuscript.

MU undertook all the MDS to Medicare claim based analyses of the match between the diagnoses as well as the cross-walk between pairs of MDS data elements, generating the appendices, tables and text for the methods and results section of the manuscript.

SC undertook all analyses of the alpha reliability analyses, prepared a first draft of the relevant methods and results sections of the manuscript and edited and reviewed all aspects of the document.

All authors read and approved of the final version of the manuscript.

## Pre-publication history

The pre-publication history for this paper can be accessed here:

http://www.biomedcentral.com/1472-6963/11/78/prepub

## Supplementary Material

Additional file 1 **Summary of information for all years of data**. Click here for file

Additional file 2 **Supplemental tables**. Click here for file
