# Supplementary material for: Temporal and Geographic variation in the validity and internal consistency of the Nursing Home Resident Assessment Minimum Data Set 2.0
Source: BMC Health Serv Res. 2011 Apr 15;11:78. doi: 10.1186/1472-6963-11-78 (PMC3097253; doi:10.1186/1472-6963-11-78)
Supplement: Additional file 1 — Summary of information for all years of data. [file 1472-6963-11-78-S1.PDF]

# ADDITIONAL FILE 1

## Sensitivity by Year

| Claims | MDS | 2000 | 2001 | 2002 | 2003 | 2004 | 2005 | 2006 |
|---|---|---|---|---|---|---|---|---|
| Any hypertension | Hypertension | 0.83 | 0.84 | 0.73 | 0.62 | 0.63 | 0.63 | 0.65 |
| Heart failure/cardiomegaly | Heart faiure | 0.68 | 0.68 | 0.68 | 0.68 | 0.68 | 0.68 | 0.67 |
| Cerebrovascular disorders | Stroke/TIA | 0.77 | 0.77 | 0.75 | 0.74 | 0.73 | 0.70 | 0.70 |
| Parkinson's disease | Parkinson's disease | 0.86 | 0.86 | 0.77 | 0.68 | 0.68 | 0.69 | 0.69 |
| Alzheimer's disease | Alzheimer's disease | 0.65 | 0.65 | 0.60 | 0.54 | 0.54 | 0.54 | 0.53 |
| Brain degeneration | Any-type dementia | 0.85 | 0.86 | 0.79 | 0.72 | 0.72 | 0.72 | 0.72 |
| Asthma/COPD/emphysema | Asthma/COPD/emphysema | 0.76 | 0.76 | 0.76 | 0.76 | 0.76 | 0.76 | 0.75 |
| Any pneumonia | Pneumonia | 0.63 | 0.62 | 0.62 | 0.62 | 0.61 | 0.62 | 0.60 |
| Depressive disorders | Depression | 0.74 | 0.75 | 0.76 | 0.77 | 0.77 | 0.77 | 0.78 |
| Diabetes mellitus | Diabetes mellitus | 0.92 | 0.92 | 0.92 | 0.92 | 0.92 | 0.93 | 0.93 |
| Any cancer | Cancer | 0.79 | 0.78 | 0.64 | 0.51 | 0.51 | 0.51 | 0.51 |
| Any anemia | Anemia | 0.49 | 0.51 | 0.45 | 0.38 | 0.38 | 0.39 | 0.39 |
| Any UT infection | Unrinary tract infection | 0.61 | 0.61 | 0.61 | 0.61 | 0.61 | 0.60 | 0.61 |
