# Supplementary material for: Temporal and Geographic variation in the validity and internal consistency of the Nursing Home Resident Assessment Minimum Data Set 2.0
Source: BMC Health Serv Res. 2011 Apr 15;11:78. doi: 10.1186/1472-6963-11-78 (PMC3097253; doi:10.1186/1472-6963-11-78)
Supplement: Additional file 2 — Supplemental tables. [file 1472-6963-11-78-S2.PDF]

ADDITIONAL FILE 2

Sensitivity by Year								
**MDS "Gold"**	**MDS Item**	**2000**	**2001**	**2002**	**2003**	**2004**	**2005**	**2006**
ADL(a) ≠ 0	Hemiplegia	0.061	0.061	0.054	0.056	0.044	0.049	0.048
ADL(a) ≠ 0	Bed-ridden	0.072	0.064	0.054	0.055	0.039	0.045	0.041
ADL(a) ≠ 0	Bed mobility = 0	0.000	0.000	0.000	0.000	0.000	0.000	0.000
ADL(a) ≠ 0	Terminal prognosis	0.028	0.028	0.025	0.023	0.015	0.018	0.017
ADL(a) ≠ 0	Pressure sore stage 3-4	0.029	0.030	0.027	0.032	0.026	0.030	0.029
CPS	Alzheimer's disease	0.101	0.110	0.096	0.097	0.087	0.102	0.101
CPS	Vascular-type dementia	0.266	0.271	0.234	0.233	0.203	0.239	0.246
Vision impairment	Cataract	0.085	0.077	0.061	0.055	0.045	0.050	0.050
Edema	No dehydration	0.979	0.984	0.985	0.989	0.988	0.992	0.993
Joint pain	Arthritis	0.420	0.429	0.403	0.417	0.375	0.434	0.442
Cancer	Chemotherapy	0.051	0.053	0.047	0.054	0.044	0.052	0.050
Any ulcer	Ulcer care	0.652	0.672	0.653	0.659	0.652	0.707	0.729
Edema	Diuretic received	0.040	0.040	0.038	0.042	0.035	0.042	0.042
